# Transarterial embolisation is associated with improved survival in patients with pelvic fracture: propensity score matching analyses

**DOI:** 10.1007/s00068-020-01497-9

**Published:** 2020-09-19

**Authors:** Hohyun Kim, Chang Ho Jeon, Jae Hun Kim, Hyun-Woo Sun, Dongyeon Ryu, Kang Ho Lee, Chan Ik Park, Jae Hoon Jang, Sung Jin Park, Seok Ran Yeom

**Affiliations:** 1 Department of Trauma and Surgical Critical Care, Pusan National University Hospital, 179 Gudeok-Ro, Seo-Gu, Busan, 602-739 Korea; 2 Biomedical Research Institute, Pusan National University Hospital, Busan, Korea; 3 Pusan National University School of Medicine, Yangsan, Korea; 4 Department of Diagnostic Radiology, Pusan National University Hospital, Busan, Korea; 5 Department of Orthopaedic Surgery, Pusan National University Hospital, Busan, Korea; 6 Department of Emergency Medicine, Pusan National University Hospital, Busan, Korea

**Keywords:** Multiple trauma, Therapeutic embolisation, Pelvic bone, Survival, Propensity score matching

## Abstract

**Introduction:**

Transarterial embolisation (TAE) is an effective intervention for management of arterial haemorrhage associated with pelvic fracture. However, its effects on survival and clinical outcomes are unclear.

**Methods:**

Trauma patients with survival data between November 2015 and December 2019 were identified using a trauma database. Patients were divided between TAE and non-TAE groups, and a propensity score was developed using multivariate logistic regression. Survival at 28 days was compared between the groups after propensity score matching.

**Results:**

Among 881 patients included in this study, 308 (35.0%) were treated with TAE. After propensity score matching, 130 pairs were selected. Survival at 28 days was significantly higher among patients treated with TAE than among those treated without TAE [122 (93.9%) vs. 112 (86.2%); odds ratio = 2.45; 95% CI 1.02–5.86; *p* = 0.039].

**Conclusions:**

TAE use was associated with improved survival at 28 days in patients with pelvic fracture and should therefore be considered in the management of severely injured patients with pelvic fracture.

## Introduction

Despite recent advances in trauma surgery, successful treatment of severe pelvic fractures remains challenging. The overall mortality of pelvic fractures is 5–10%, increasing to 60% in patients with haemodynamic instability [1–3]. Bleeding is the major cause of death after severe pelvic fracture; the origin of the bleeding can be arterial, venous, or bone-related [4]. Arterial bleeding produces the most severe haemorrhage and frequently results in hypotension [5]. Rapid control of haemorrhage is associated with improved survival [6].

Pelvic angiography and subsequent embolisation is a safe, rapid, and effective technique for patients with pelvic fracture-related arterial haemorrhage in both haemodynamically stable and unstable patients [7–9]. Transarterial embolisation (TAE) is the mainstay of treatment for arterial bleeding. However, there is no consensus as to the optimal treatment paradigm for patients presenting with severe pelvic fracture [6, 10].

It is difficult to assess the outcome of TAE, as a comparison between published series is hampered by differences in patient populations and because the outcome is often determined by factors other than pelvic haemorrhage alone, such as associated traumatic injuries. No randomised controlled trials have been conducted, and it is unlikely that any will be performed.

Accordingly, in an effort to verify the efficacy of TAE in patients with pelvic injury, we examined outcomes in patients treated with TAE compared to those treated without TAE. We used propensity score matching analyses as the most reliable method to reduce the effects of confounding factors in this retrospective study. We hypothesised that TAE would improve survival at 28 days in trauma victims.

## Methods

### Study setting

**Fig. 1:**
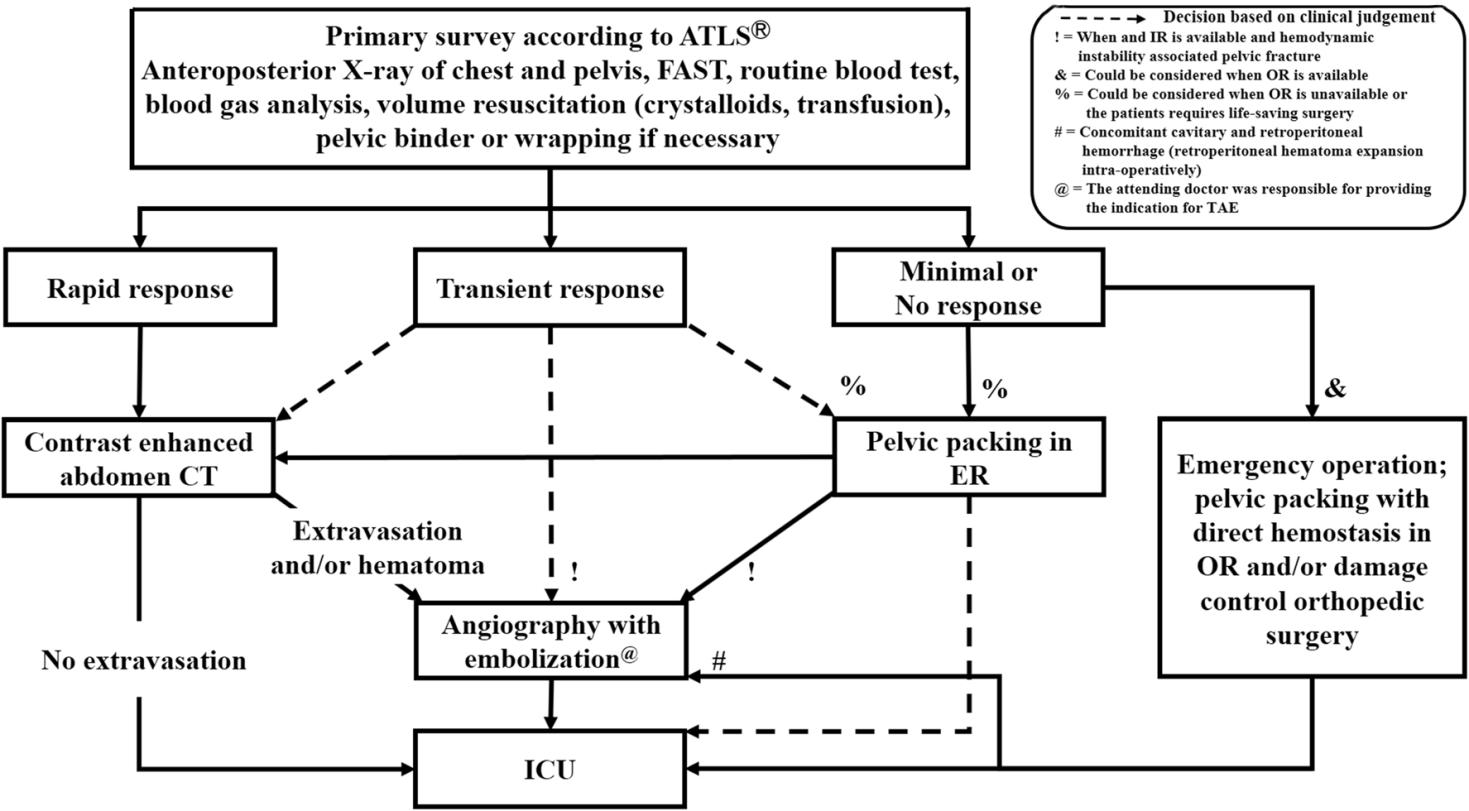
Pelvic fracture management algorithm. *ATLS* adult trauma life support, *FAST* Focused Assessment with Sonography in Trauma, *ER* emergency room, *IR* interventional room, *OR* operating room, *ICU* Intensive Care Unit

Pusan National University Hospital Regional Trauma Center is the largest trauma centre in Korea, established as a regional Level I Trauma Center. Annually, there are more than 2500–3000 trauma-related admissions, of which 900–1000 patients present with major trauma. The centre is equipped with a trauma bay, a 42-bed dedicated trauma intensive care unit (ICU), and a trauma angiography suite. Three interventional radiologists and the equipment required for TAE are available 24 h a day, 7 days a week. Thus, the time from arrival to TAE can be < 2 h. Patients with pelvic fractures without other extrapelvic injuries requiring emergency treatment are treated according to the pelvic fracture management algorithm (Fig. 1). The indications for pelvic TAE are (1) contrast media extravasation or expanding peripelvic haematoma in contrast-enhanced abdominopelvic computed tomography (CT) or (2) persistent haemodynamic instability associated with pelvic fracture without another significant source of bleeding even if extravasation is not seen on CT. The contraindications for pelvic TAE include minimal or non-responders.

### Study population

Pelvic injuries almost always accompany injuries to other organ systems. Considering pelvic injuries in isolation would not be realistic; thus, polytrauma patients with pelvic bone fracture were included in this study. We retrospectively reviewed data from the medical records and included a total of 1017 patients with pelvic fracture admitted to the trauma resuscitation unit (TRU) at our trauma centre between November 2015 and December 2019.

Available data included age, sex, mechanism of injury, vital signs on arrival, packed red blood cells transfusion within 4 and 24 h after arrival, Abbreviated Injury Scale (AIS) score, Injury Severity Score (ISS), shock index, Trauma Related Injury Severity Score (TRISS) score, massive transfusion within the initial 24 h after arrival, length of hospital stay, ICU stay, and survival status at 7 days, 28 days, and discharge. Pelvic fractures were defined as pelvic ring or acetabular fractures. Details of the pelvic ring fractures were recorded, including pelvic injury AIS and fracture type (A, B, or C type) according to the Tile classification [11] for pelvic fractures. The shock index was defined as heart rate (beat/min)/systolic blood pressure (SBP; mmHg). Persistent haemodynamic instability was defined as persistent hypotension (SBP < 90 mmHg) in spite of loading 2 L of crystalloid and transfusion of 2 units of packed red blood cells. Massive transfusion was defined as the replacement by transfusion of 10 units of red cells in 24 h.

### Outcome measures

The primary outcome was survival at 28 days. Secondary outcomes included survival at 7 days, survival-to-discharge, hospital-free days to day 90, and ICU-free days to day 28. Hospital-free days to day 90 is a composite of in-hospital death and hospital length of stay, defined as the number of days alive and out of the hospital between the hospital arrival and 90 days later. Patients who died during the index hospitalisation and those hospitalised for > 90 days were classified as having zero hospital-free days. For patients discharged alive before day 90, hospital-free days were calculated as 90 minus the length of stay. ICU-free days to day 28 were calculated in the same way as hospital-free days to day 90.

### Statistical analyses

Patient data were divided between TAE and non-TAE groups. The TAE group consisted of patients who were treated with TAE in conjunction with other standard resuscitation and haemostasis procedures in the TRU, while the non-TAE group consisted of those who were treated with standard care without TAE.

Because many cofounders can affect survival-to-discharge, such as vital signs on presentation, severity of injuries, and procedures for definitive haemostasis, propensity score matching was performed to compare the primary outcome between both groups, as well as to assess secondary outcomes. A propensity score was developed using logistic regression to estimate the probability of being assigned to the TAE group compared to the non-TAE group. Relevant covariates were carefully selected from known or possible survival predictors in trauma victims and were entered into the propensity model to ensure high-fidelity propensity scores. Patients with missing covariates were excluded from propensity score calculation. The precision of discrimination and propensity score calibration were analysed with the C-statistic and Hosmer–Lemeshow goodness-of-fit tests. Then a one-to-one propensity score matching was performed using a greedy matching algorithm without replacement, where a calliper width of < 0.001 in the logit-transformed propensity score was applied. The inter-group comparison of the primary outcome after propensity score matching was performed using linear regression analyses.

In addition to comparing survival at 28 days between the TAE and non-TAE groups, Kaplan–Meier plots of survival curves up to 28 days for each group were drawn. Hazard ratios were calculated using a proportional hazard model.

Several subgroup analyses were also performed to evaluate the heterogeneity of the treatment effect of TAE. One of the subgroups selected included patients with a high-grade pelvic injury, defined as patients who had an AIS ≥ 4 or a Tile classification of C in the pelvis. Primary and secondary outcomes were compared between the TAE and non-TAE groups in the selected patients after propensity score matching.

Summary statistics are reported as medians where appropriate. The Mann–Whitney *U* test and the Wilcoxon rank sum test were used for comparisons of the median values of continuous variables and for ordinal data, respectively, whereas the Chi-square test and Fisher’s exact test were used to compare frequencies of categorical variables between groups. A value of *p* < 0.05 was considered statistically significant. The Statistical Package for the Social Sciences (Version 20.0, SPSS, Inc., Chicago, IL, USA) and STATA (Version 14.2, Stata Corp., College Station, TX, USA) were used to analyse the data.

## Results

### Patient characteristics

**Fig. 2:**
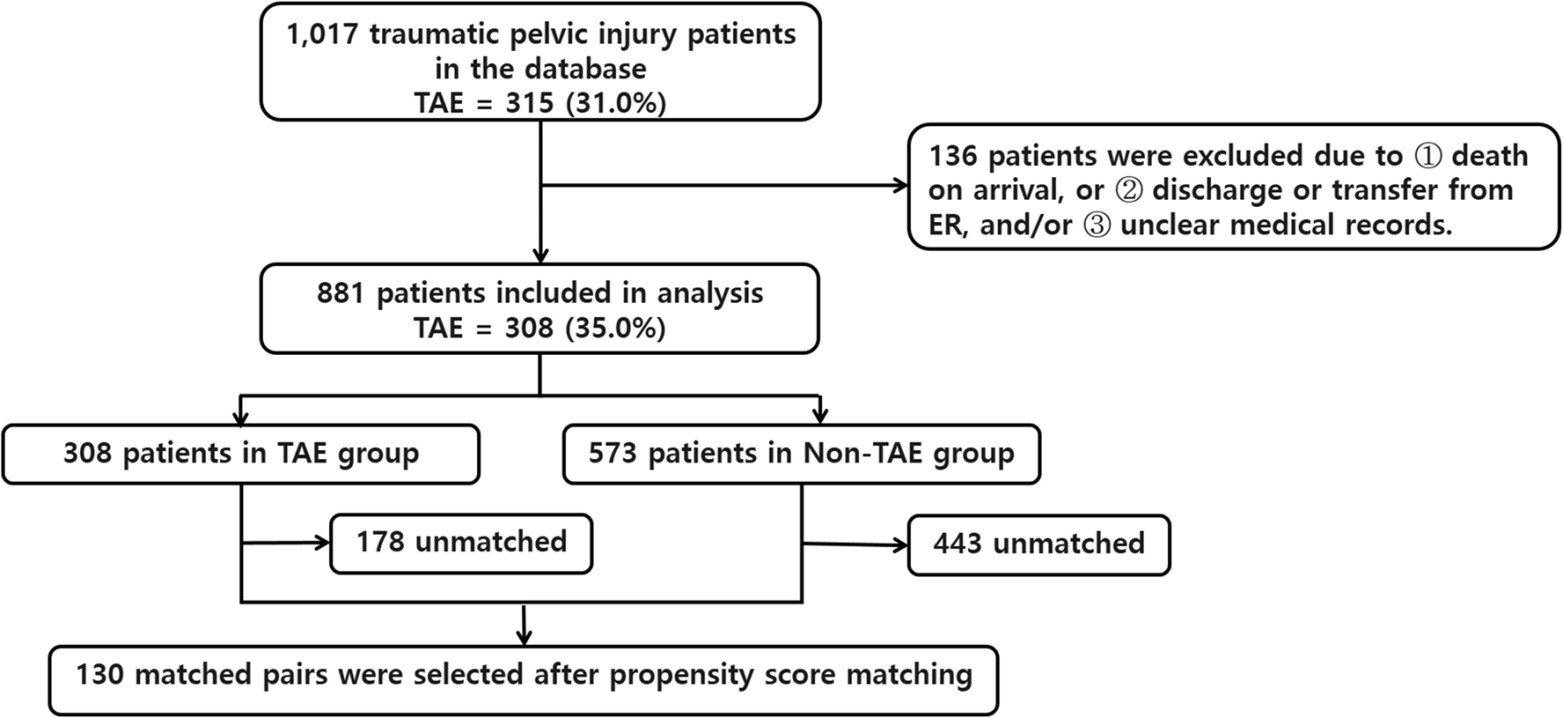
Flowchart of the study. *TAE* transarterial embolisation

After screening, 1017 traumatic pelvic injury patients were identified for inclusion in the study. Of these, 315 (31.0%) patients underwent TAE treatment in conjunction with other standard resuscitation. Another 136 were excluded due to being declared dead-on-arrival and/or discharge or transfer from TRU within 1 day and/or unclear medical records. The patient flow diagram is summarised in Fig. 2.

A total of 881 patients were eligible for this study, among whom 308 (35.0%) were treated with TAE and 573 (65.0%) were not. Overall mortality was 15%. Haemodynamic instability (shock index ≥ 0.9) was present on admission in 489 patients (55.5%). The median time to embolisation was 107 min (interquartile range, 80–142). Patient characteristics are summarised in Table 1. Patients in the TAE group had significantly lower SBP and a higher shock index on arrival compared to those in the non-TAE group [90 (70–110) vs. 100 (80–120) and 1.0 (0.7–1.4) vs. 0.9 (0.7–1.2), respectively], as well as a higher ISS [29 (22–38) vs. 24 (21–33)], higher lactic acid level [3.8 (2.4–6.0) vs. 3.2 (2.0–5.3)], lower base excess [ − 3.9 ( − 7.6–0.7) vs. -2.2 ( − 6.3–0.5)], and lower TRISS-calculated probability of survival (Ps) [0.88 (0.65–0.94) vs. 0.93 (0.78–0.96)]. Furthermore, more patients in the TAE group required pelvic surgery and transfusion within 24 h after arrival. We found no differences in the ratio of patients who were treated with pelvic stabilisation (external fixation) and preperitoneal packing (PPP) between the TAE and non-TAE groups.

**Table 1:** Characteristics of patients treated with or without transarterial embolisation

Variable	Before matching			After matching		
	TAE	Non-TAE	*p* value	TAE	Non-TAE	*p* value
Case	308	573		130	130	
Age (years)	58 (42–73)	55 (37–67)	0.004	58 (40–68)	54 (39–66)	0.191
Female (%)	126 (40.9)	174 (30.4)	0.002	40 (30.1)	52 (40.0)	0.120
Injury mechanism (%)			< 0.001			0.297
Car TA	21 (6.8)	103 (18.0)		11 (8.5)	24 (18.5)	
Motorcycle TA	33 (10.7)	52 (9.1)		17 (13.1)	12 (9.2)	
Pedestrian TA	112 (36.4)	166 (29.0)		42 (32.3)	37 (28.5)	
Fall	108 (35.1)	195 (34.0)		51 (39.2)	48 (36.9)	
Entrapment	15 (4.9)	22 (3.8)		5 (3.9)	5 (3.9)	
Others	19 (6.2)	35 (6.1)		4 (3.1)	4 (3.1)	
SBP (mmHg)	90 (70–110)	100 (80–120)	< 0.001	100 (80–110)	100 (80–120)	0.694
Heart rate (beats/min)	95 (81–114)	95 (80–112)	0.788	95 (83–112)	96 (81–110)	0.933
Shock index	1.0 (0.7–1.4)	0.9 (0.7–1.2)	< 0.001	1.0 (0.8–1.3)	0.9 (0.8–1.3)	0.452
Shock index ≧ 0.9	198 (64.3)	291 (50.8)	< 0.001	77 (59.2)	76 (58.5)	0.900
Lactic acid (mmol/L)	3.8 (2.4–6.0)	3.2 (2.0–5.3)	0.005	3.3 (2.2–5.0)	3.2 (2.0–5.6)	0.503
Base excess	− 3.9 ( − 7.6–0.7)	− 2.2 ( − 6.3–0.5)	0.001	− 2.7 ( − 5.6–0.2)	− 2.9 ( − 7.0–0.9)	0.996
ISS	29 (22–38)	24 (21–33)	< 0.001	27 (22–34)	27 (21–34)	0.715
GCS	15 (12–15)	15 (10–15)	0.647	15 (13–15)	15 (12–15)	0.920
Pelvic injury AIS			< 0.001			0.825
2	102 (33.1)	386 (67.4)		67 (51.5)	63 (48.5)	
3	27 (8.8)	51 (8.9)		15 (11.5)	13 (10.0)	
4	111 (36.0)	112 (19.5)		39 (30.0)	46 (35.4)	
5	68 (22.1)	24 (4.2)		9 (6.9)	8 (6.1)	
Tile classification			< 0.001			0.850
A	102 (33.1)	386 (67.4)		67 (51.5)	63 (48.5)	
B	138 (44.8)	163 (28.3)		54 (41.5)	59 (45.4)	
C	68 (22.1)	24 (4.2)		9 (6.9)	8 (6.1)	
Head and neck AIS ≧ 4	40 (13.0)	109 (19.0)	0.023	19 (14.6)	21 (16.2)	0.731
Chest AIS ≧ 4	36 (11.7)	102 (17.8)	0.017	16 (12.3)	25 (19.2)	0.126
TRISS score	0.88 (0.65–0.94)	0.93 (0.78–0.96)	< 0.001	0.91 (0.78–0.95)	0.92 (0.80–0.96)	0.169
pRBC consumption within 24 h	4 (2–11)	2 (0–5)	< 0.001	3 (2–6)	3 (0–6)	0.065
MT within 24 h	85 (27.6)	77 (13.4)	< 0.001	22 (16.9)	17 (13.1)	0.385
Surgery	264 (85.7)	455 (79.4)	0.021	113 (86.9)	113 (86.9)	1.000
Surgery within 24 h	110 (35.8)	199 (34.7)	0.744	42 (32.3)	51 (39.2)	0.244
Pelvic surgery within 24 h	26 (8.4)	18 (3.1)	0.001	7 (5.4)	8 (6.2)	0.790
EF^a^	7 (26.9)	3 (16.7)	0.331	1 (14.3)	1 (12.5)	0.919
ORIF^a^	19 (73.1)	15 (83.3)		6 (85.7)	7 (87.5)	
PPP	6 (1.9)	9 (1.6)	0.675	2 (1.5)	2 (1.5)	1.000
Referral from scene (%)	148 (48.1)	280 (48.9)	0.818	63 (48.5)	62 (47.7)	0.901

Values are presented as numbers (%) or medians (range)

*TAE* transarterial embolisation, *TA* traffic accident, *SBP* systolic blood pressure, *ISS* Injury Severity Score, *GCS* Glasgow Coma Scale, *AIS*, Abbreviated Injury Scale, *TRISS* Trauma Related Injury Severity Score, *pRBC* packed red blood cells, *MT* massive transfusion, *EF* external fixation, *ORIF* open reduction and internal fixation, *PPP* preperitoneal packing

^a^Attributable percentage of pelvic surgery within 24 h

### Propensity score matching

Considering the non-negligible biased distributions in known survival predictors of trauma patients, propensity score matching was performed. The final propensity model predicting allocation to the TAE group included as covariates age, sex, vital signs on arrival (GCS, heart rate, SBP, and shock index), mechanism of injury, lactic acid level and base excess, pelvic bone injury AIS, performance of a haemostatic procedure (pelvic stabilisation [external fixation] and/or PPP), massive transfusion within the initial 24 h after arrival, ISS, and TRISS-calculated probability of survival. This model was validated to have high discrimination and calibration for the probability of being assigned to the TAE group (C-statistic = 0.972 and Hosmer–Lemeshow goodness of fit *p* = 0.821).

### Impact of TAE on survival at 28 days and secondary outcomes

Among the 308 patients in the TAE group, 130 patients were matched with patients in the non-TAE group. Patient characteristics after matching are summarised in Table 1. Propensity score matching revealed that overall survival-to-discharge was no different between the TAE and non-TAE groups [120 (92.3%) vs. 111(85.4%); *p* = 0.076]. However, survival at 28 days was significantly higher among patients treated with TAE than among those treated without it [122 (93.9%) vs. 112 (86.2%); odds ratio (OR) = 2.45; 95% confidence interval (CI) 1.02–5.86; *p* = 0.039; Table 2]. TAE use was associated with improved survival at 7 and 28 days after injury (OR = 3.02; 95% CI 1.05–8.64; *p* = 0.032; Table 2). ICU-free days to day 28, and hospital-free days to day 90 in these patients are also shown in Table 2.

**Table 2:** Clinical outcomes of patients treated with or without TAE

Variable	After matching				
	TAE (*n* = 130)	Non-TAE (*n* = 130)	*p *value	OR	95% CI
Survival at 28 days (%)	122 (93.9)	112 (86.2)	0.039	2.45	1.02–5.86
Survival at 7 days (%)	125 (96.2)	116 (89.2)	0.032	3.02	1.05–8.64
Survival-to-discharge (%)	120 (92.3)	111 (85.4)	0.076	2.05	0.92–4.61
Hospital-free days to day 90 (days)	59 (22–67)	55 (30–70)	0.990		
ICU-free days to day 28 (days)	24 (12–26)	23 (14–27)	0.473		

Values are presented as numbers (%) or medians (range)

*TAE* transarterial embolisation, *OR* odds ratio, *CI* confidence interval, *ICU* Intensive Care Unit

**Fig. 3:**
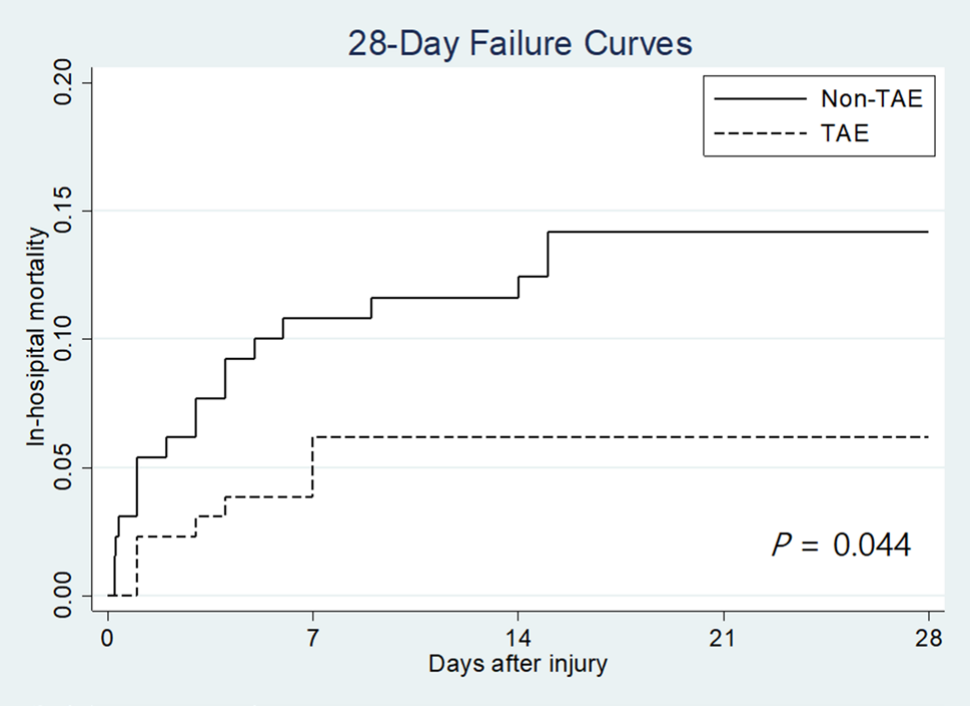
Kaplan–Meier 28-day failure curves of patients treated with transarterial embolisation (TAE) or without TAE. Failure to survive at 28 days was significantly lower in patients in the TAE group than those in the non-TAE group [hazard ratio (HR) = 0.42; 95% CI 0.18–0.98; *p* = 0.044]

**Fig. 4:**
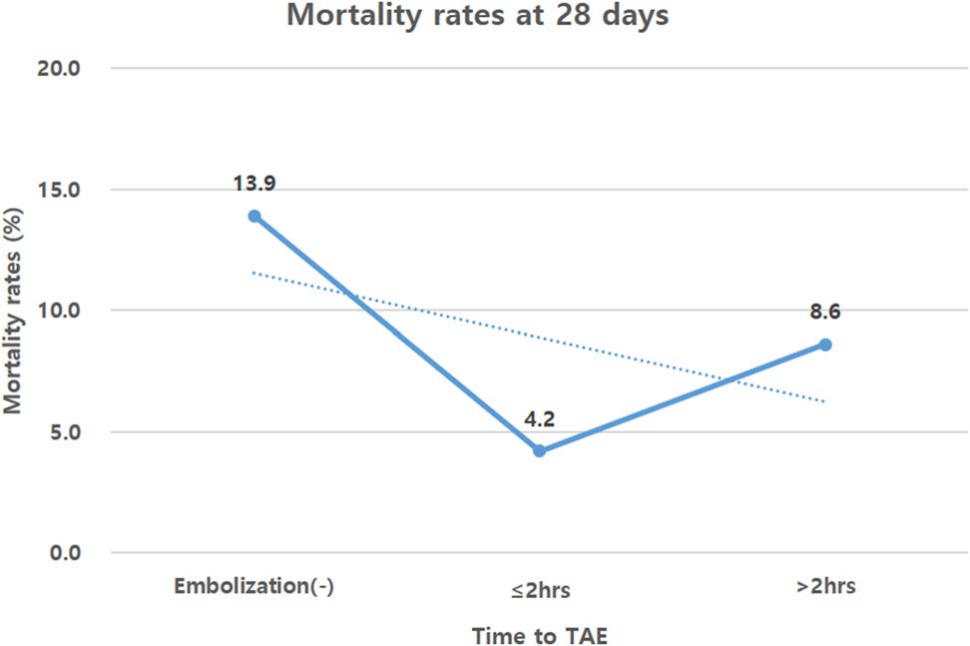
Relationship between mortality rates at 28 days and the time to transarterial embolisation (TAE). Performing TAE < 2 h after admission led to better survival at 28 days than non-TAE (OR = 3.70; 95% CI 1.05–13.01; *p* = 0.042)

Kaplan–Meier plots of failure curves for patients treated with and without TAE are shown in Fig. 3. Failure to survive at 28 days was significantly lower in patients in the TAE group than those in the non-TAE group [hazard ratio (HR) = 0.42; 95% CI 0.18–0.98; *p* = 0.044]. Figure 4 demonstrates the relationship between mortality rates at 28 days and the time to TAE. Performing TAE < 2 h after admission led to better survival at 28 days than non-TAE (OR = 3.70; 95% CI 1.05–13.01; *p* = 0.042).

ICU-free days to day 28 and hospital-free days to day 90 did not differ significantly between the TAE and non-TAE groups [24 [12–26] days vs. 23 [14–27] days; *p* = 0.473 and 59 (22–67) days vs. 55 (30–70) days; *p* = 0.990, respectively; Table 2].

### Subgroup analyses

Subgroup analyses performed to evaluate the heterogeneity of the treatment effect of TAE identified that TAE was significantly associated with improved survival at 28 days in patients with a high-grade pelvic injury [45 (93.8%) in the TAE group vs. 43 (79.6%) in the non-TAE group; OR = 3.84; 95% CI 1.00–14.70; *p* = 0.039; Table 3]. Survival at 7 days was also significantly higher in patients in the TAE group than those in the non-TAE group (OR = 9.40; 95% CI 1.14–77.22; *p* = 0.013; Table 3).

**Table 3:** Effectiveness of TAE in patients with high-grade pelvic injury

Variable	After matching				
	TAE (*n* = 48)	Non-TAE (*n* = 54)	*p *value	OR	95% CI
Survival at 28 days (%)	45 (93.8)	43 (79.6)	0.039	3.84	1.00–14.70
Survival at 7 days (%)	47 (97.9)	45 (83.3)	0.013	9.40	1.14–77.22
Survival-to-discharge (%)	44 (91.7)	43 (79.6)	0.087	2.81	0.83–9.52
Hospital-free days to day 90 (days)	58 (28–65)	49 (0–62)	0.225		
ICU-free days to day 28 (days)	25 (12–27)	23 (15–26)	0.502		

Values are presented as numbers (%) or medians (range)

*TAE* transarterial embolisation, *OR* odds ratio, *CI* confidence interval, *ICU* Intensive Care Unit

## Discussion

Propensity score matching indicated that TAE is independently associated with improved survival at 28 days in trauma patients with pelvic fracture. To the best of our knowledge, this is the first study to report this relationship using robust statistical methods. Notably, the observed relationship was consistent in the survival at 7 days, and a significantly lower hazard ratio of death from TAE was detected among patients.

Hauschild et al. [12] reported no significant difference in overall mortality rate when comparing the TAE group with the non-embolisation group. However, several studies have demonstrated that control of pelvic arterial haemorrhage using TAE was associated with a 7–35% reduction in mortality [7, 9, 13]. Furthermore, some studies have reported the occurrence of arterial bleeding on angiography in patients with pelvic fracture even in the absence of contrast media extravasation on CT [8, 10, 14]. Non-TAE group patients may have arterial injury even without contrast media extravasation on the CT, and this bleeding, if continuous, may affect the outcome. We therefore believe that the use of TAE should be considered in the management of severely injured patients with pelvic fracture.

Pelvic fractures are often caused by high-energy events. Haemodynamically unstable patients who present with traumatic pelvic fracture are at high risk for mortality and significant morbidity [15, 16]. The mortality rate of pelvic fractures is < 20%, but that of severe pelvic fracture combined with multiple injuries is as high as 30–70%, and the prognosis is poor [12, 17–20]. In our study, overall mortality was 15% and the mortality of those presenting with haemodynamic instability and severe pelvic injury is similar to the mortality rates in other published series [21, 22].

Most patients with severe pelvic fracture died due to haemorrhage or sepsis/multiple organ failure [21]. The cause of early mortality was often haemorrhage, whereas the cause of late mortality was sepsis/organ failure or traumatic brain injury. Thus, we did not choose survival-to-discharge, but instead survival at 28 days as a primary outcome to distinguish deaths due to bleeding from others. In our propensity matching data, sepsis and organ failure accounted for 3 patients who died after 28 days. We think this explains why overall survival-to-discharge was no different between the TAE and the non-TAE group (*p* = 0.076). However, survival at 7 and 28 days were significantly higher among patients treated with TAE than among those treated without TAE (*p* = 0.032; *p* = 0.039, respectively).

The bony surfaces within a pelvic fracture can cause haemorrhage via arterial and/or venous injury or from the fractured bone itself [18]. Therefore, a multidisciplinary approach using various haemorrhage control modalities is necessary. The overwhelming majority of pelvic bleeding is of venous origin [23]. However, arterial bleeding, while less common than venous bleeding in the pelvis, is more common in patients that are persistently hypotensive. Some have reported that pelvic arterial bleeding is found in > 70% of pelvic fracture patients with transient or no response to fluid resuscitation [8, 14]. TAE is currently accepted in many trauma centres as the preferred method for controlling pelvic arterial haemorrhage [7, 8, 14, 21, 24–26].

In this study, pelvic TAE was performed in 35% of all patients presenting with pelvic fracture. In other studies, 3.8–9.6% of patients with pelvic fractures required pelvic TAE [18, 22, 27]. Even though there is no contrast media extravasation on CT, patients with haemodynamic instability associated with pelvic fracture without another significant source of bleeding may have arterial bleeding on angiography [8, 10, 14]. Therefore, at our centre, angiography was performed when the expanding hematoma was visible or if there was persistent haemodynamic instability associated with the pelvic fracture even in the absence of extravasation on CT. Our interventional radiologists and the equipment required for TAE are available around the clock, with the attending doctor being responsible for providing the indication for TAE (Fig. 1). We believe this is the reason for the increased proportion of TAE in the present study.

A recent study reported that every hour of delay in pelvic TAE is associated with an increased risk for in-hospital death by 79% [28]. Balogh et al. noted that patients with pelvic fractures and unstable haemodynamics should receive TAE within 90 min after admission, as this reduces blood transfusion volumes and mortality [29]. Any delay exposes patients to unnecessary risks [28–30]. Therefore, early access to angiography is associated with reduced mortality. Delays to embolisation > 3 h are associated with worse outcomes [21]. In our study, the median time to TAE was 107 min; performing TAE < 2 h after admission led to better survival at 28 days than non-TAE (Fig. 4).

There are two main differences between our study and other studies that suggest that TAE might be useful. First, the number of covariates included in the propensity score calculation was higher than in other studies [14, 22, 25]. We considered that outcome predictors such as the performance of a haemostatic procedure (pelvic stabilisation [external fixation] and/or PPP) or massive transfusion within the initial 24 h after arrival should be entered into the propensity model regardless of their relevance to TAE, because biased distribution of these factors would significantly affect survival. Second, we performed matching through a greedy matching algorithm with a calliper width of less than 0.001 in the logit-transformed propensity score. Accordingly, only 130 (24.2%) patients in the TAE group were matched with those in the non-TAE group. Because the deliberate selection of covariates and the strict matching algorithm could make patient characteristics of the TAE and non-TAE groups more similar, including TRISS-calculated Ps (0.91 in the TAE group vs. 0.92 in the non-TAE group; Table 1), the significant association between TAE and improved survival found in our study suggests that TAE could be beneficial in injured patients with pelvic fracture.

There were several limitations to this study. First, because it was retrospective, the results are not conclusive. Although we demonstrated a higher survival rate at 28 days in patients with pelvic fracture treated with TAE than in those treated without TAE, residual confounding and unmeasured survival predictors could exist as impediments to confirming the efficacy of TAE. Further clinical investigations, including a randomised controlled trial, are needed to validate our results.

Another limitation is the fact that 178 (57.8%) of patients in the TAE group were excluded from the propensity score calculation or matching process, which may limit the generalisability of our findings. The association between TAE and improved survival might not be applicable to patients who were excluded during propensity model development nor in patients outside the database. Although limited in scope, considering that the patient characteristics of the TAE group in this study (in-hospital mortality was 11.2%, median ISS was 27, and median base excess was  − 2.8) were similar to the populations reported in other studies [17, 18, 21], we believe our results might be applicable to severely injured trauma victims around the world.

Finally, this study was confined to patients at a single centre and our study population may not represent all patients with pelvic fracture. For example, our TRISS-calculated Ps are lower than in other studies [23, 31]. This is because patients who were declared dead-on-arrival and/or discharged or transferred from TRU within 24 h were excluded from our study. Therefore, a larger group of patients and further studies (more rigorous research design and external validation) are necessary to draw any definitive conclusions.

## Conclusion

In severely injured trauma patients with pelvic injury, TAE is associated with improved survival at 7 and 28 days after injury. The use of TAE should therefore be considered in conjunction with trauma resuscitation during the management of patients with severe pelvic fractures.
